# The role of community health worker-based care in post-conflict settings: a systematic review

**DOI:** 10.1093/heapol/czac072

**Published:** 2022-09-19

**Authors:** Kalin Werner, Mohini Kak, Christopher H Herbst, Tracy Kuo Lin

**Affiliations:** Institute for Health and Aging, Department of Social and Behavioral Sciences, University of California, 490 Illinois Street, 12th Floor, Box 0646, San Francisco, CA 94158, USA; Division of Emergency Medicine, University of Cape Town, F51 Old Main Building, Groote Schuur Hospital, Observatory, Cape Town 7935, South Africa; Health, Nutrition and Population Global Practice, The World Bank, 1818 H Street, N.W., Washington, DC 20433, USA; Health, Nutrition and Population Global Practice, The World Bank, 1818 H Street, N.W., Washington, DC 20433, USA; Institute for Health and Aging, Department of Social and Behavioral Sciences, University of California, 490 Illinois Street, 12th Floor, Box 0646, San Francisco, CA 94158, USA

**Keywords:** Post-conflict, community health, community health workers, community-based health care, systematic reviews, *CHW-based care in post-conflict settings*

## Abstract

Countries affected by conflict often experience the deterioration of health system infrastructure and weaken service delivery. Evidence suggests that healthcare services that leverage local community dynamics may ameliorate health system-related challenges; however, little is known about implementing these interventions in contexts where formal delivery of care is hampered subsequent to conflict. We reviewed the evidence on community health worker (CHW)-delivered healthcare in conflict-affected settings and synthesized reported information on the effectiveness of interventions and characteristics of care delivery. We conducted a systematic review of studies in OVID MedLine, Web of Science, Embase, Scopus, The Cumulative Index to Nursing and Allied Health Literature (CINHAL) and Google Scholar databases. Included studies (1) described a context that is post-conflict, conflict-affected or impacted by war or crisis; (2) examined the delivery of healthcare by CHWs in the community; (3) reported a specific outcome connected to CHWs or community-based healthcare; (4) were available in English, Spanish or French and (5) were published between 1 January 2000 and 6 May 2021. We identified 1976 articles, of which 55 met the inclusion criteria. Nineteen countries were represented, and five categories of disease were assessed. Evidence suggests that CHW interventions not only may be effective but also efficient in circumventing the barriers associated with access to care in conflict-affected areas. CHWs may leverage their physical proximity and social connection to the community they serve to improve care by facilitating access to care, strengthening disease detection and improving adherence to care. Specifically, case management (e.g. integrated community case management) was documented to be effective in improving a wide range of health outcomes and should be considered as a strategy to reduce barrier to access in hard-to-reach areas. Furthermore, task-sharing strategies have been emphasized as a common mechanism for incorporating CHWs into health systems.

Key messages

- This review characterizes the impact of community health worker (CHW)-delivered healthcare in fragile and conflict-affected settings presented in published studies.
- Evidence points to the value of leveraging CHWs to deliver healthcare and serve the unique health needs of populations residing in conflict-affected settings.
- Community-based care addresses key barriers in four critical ways: (1) increasing access to essential healthcare services, (2) improving case management and treatment adherence, (3) enhancing disease detection and monitoring and (4) facilitating the scaling up of services.
- Key enablers for community-based care include training and support provided to CHWs and material resources provided to CHWs.

## Introduction

The prevalence of armed conflict and violence globally has resulted in over 484 million people—including some of the world’s most vulnerable populations—currently living in fragile or conflict-affected states (Gleditsch *et al.*, 2016; World Health Organization, 2017; The World Bank, 2018; Institute for Economics & Peace, 2021). By 2030, it is expected that 46% of the global poor will reside in areas characterized as either fragile or conflict-affected (The World Bank, 2019). Although battle-related deaths have declined since 1946, conflict is estimated to constitute 80% of all humanitarian needs (The World Bank, 2019).

Over recent decades, a rise in protracted and recurring conflict, with frequent relapses into violence, reveals a lack of clarity for determining when states are in a period of active conflict or immediately after conflict. There is little consensus in the literature regarding the use of the term ‘post-conflict’. Fundamentally, the post-conflict period exists at a time between a conflict and peace. On the one hand, some studies identify this period as a transitional state, ‘most crucial in supporting or underpinning still fragile cease-fires or peace processes by helping to create conditions for political stability, security, justice and social equity’ (UNDG/ECHA Working Group on Transition, 2004). On the other hand, development partners and international agencies identify this period by its characteristics rather than by time. Here, post-conflict settings are identified by their ‘low levels of capability to implement core responsibilities’ (Woolcock, 2014), or ‘fundamental failure of the state to perform functions necessary to meet citizens’ basic needs and expectations’ (Department for International Development, 2005). The World Bank, well known for its annual country classifications, associates ‘high levels of institutional and social fragility’ with those categorized as fragile and conflict-affected situations.

Regardless of varying characterizations, it is clear that countries that have suffered from recent conflict, or that are now suffering from conflict, often experience a deterioration of their health system infrastructure and a weakening of service delivery. As such, populations residing in fragile, conflict-affected and post-conflict states (FCAPCS) face mass displacement and exposure to atrocities of violence, which directly increases vulnerability to communicable diseases and the prevalence of psychological disorders (Rutherford and Saleh, 2019). Disintegrated health infrastructure makes responding to the health needs of these populations even more challenging. Damaged infrastructure, limited human resources and fragmented service delivery result in substantial increases in morbidity and mortality from non-violent causes. All the while, individuals who have experienced conflict exhibit some of the worst indicators of maternal and infant mortality and need a tremendous amount of care. The populations residing in FCAPCS face enormous barriers in achieving global targets for their health systems. For example, only one in five fragile and conflict-affected states are on track to achieve the sustainable development goals (Samman *et al.*, 2018).

There is growing interest in addressing these obstacles and testing solutions to reach difficult-to-access areas in FCAPCS using low-cost, scalable solutions to offset the burden of damaged facilities, broken supply chains and weakened health workforces. Healthcare services that leverage local community dynamics pose a potential solution in contexts where formal delivery of care is hampered by conflict. Central to such services are community health workers (CHWs) or lay individuals who not only have an in-depth understanding of the community culture and language but who also serve as an effective option to address the dwindling health workforce that resulted from conflict-induced attrition (Olaniran *et al.*, 2017). CHWs are expected to undertake a number of functions such as performing health assessments, delivering remote primary care, monitoring patients for follow-up and providing targeted health education. Often this involves personalized care through case management and care coordination. CHWs also play non-therapeutic roles, providing social support, helping patients to access local services and supporting patients understand medical advice and recommendations (Hartzler *et al.*, 2018). CHWs are effective in improving population health across diverse settings (Perry and Zulliger, 2012; Perry *et al.*, 2014), have a positive impact on health development goals (Bhutta *et al.*, 2010) and are cost-effective (Vaughan *et al.*, 2015). Prior systematic reviews have further identified the value of CHWs for specific conditions, such as maternal and child health (Gilmore and Mcauliffe, 2013) or infectious disease. A growing body of literature has determined the requirements for the sustainability and scale up of CHWs in low-income settings (Pallas *et al.*, 2013). However, not enough is known about the delivery and success of CHW-based interventions in relation to the specific contextual challenges faced by post-conflict settings.

Strategies to deliver care outside a hospital or clinic may be a particularly relevant approach where disease burdens are high, but infrastructure is weakened. CHW programmes may play a critical role in linking relief, rehabilitation and development approaches, which aim to link short-term measures to longer-term development programmes for a more sustainable response to health systems under stress (Nicholls *et al.*, 2015; Affun-Adegbulu *et al.*, 2018; Miller *et al.*, 2020). However, healthcare delivery strategies that are insensitive to the fragile and conflict-affected settings risk aggravating existing disparities even further. Thus, it is critical for policymakers to understand which types of policies should be adopted in conjunction with CHW deployment to best meet the healthcare needs of the population in FCAPCS. The aim of this study is to characterize systematically the literature on the role of CHW-delivered healthcare in fragile and conflict-affected settings and synthesize reported information on the effect of these interventions on key healthcare functions.

## Methods

### Search strategy

A systematic literature review was performed following Preferred Reporting Items for Systematic Reviews and Meta-Analyses ( PRISMA) guidelines to identify articles relevant to the study topic (Liberati *et al.*, 2009). OVID MedLine, Web of Science, Embase, Scopus, The Cumulative Index to Nursing and Allied Health Literature (CINHAL) and Google Scholar databases were searched in May 2021. The search strategy used context-specific keywords—‘post conflict’, ‘post war’ and ‘fragile state’—in combination with topic-related keywords associated with CHWs and community-based healthcare (CBHC) services. The full search strategy, which combines relevant keywords using Boolean operators, can be found in Supplementary 1.

### Eligibility criteria

Articles were included if they (1) described a context that is post-conflict, conflict-affected or was impacted by war or crisis; (2) examined the delivery of healthcare by CHWs in the community; (3) reported a specific outcome connected to CHWs or CBHC; (4) were available in English, Spanish, or French and (5) were published between 1 January 2000 and 6 May 2021. We restricted our review to the most recent two decades to capture data best equipped to inform contemporary decision-making. Details of both inclusion and exclusion criteria are outlined in Table 1.

**Table 1.** Inclusion and exclusion criteria

Inclusion	Exclusion
Study context that is post-conflict, conflict-affected or impacted by war or crisis Care interventions delivered by CHWs Care delivered in community-based setting CHW- or CBHC-related outcome In English, Spanish or French Published between 1 January 2000 and 6 May 2021	A study context that is not post-conflict, conflict-affected or impacted by war or crisis Care interventions delivered by formal health professionals (e.g. doctors or nurses) Care delivered not delivered in community-based setting (e.g. in hospital) Outcome not related to CHW or CBHC In a language other than English, Spanish or French Published outside of 1 January 2000 to 6 May 2021

Box 1. Key definitions used in this review

**Table:** 

Term	Definition
CHWs	Paraprofessionals or lay individuals with an in-depth understanding of the community culture and language have received standardized job-related training of a shorter duration than health professionals; their primary goal is to provide culturally appropriate health services to the community (Olaniran *et al.*, 2017)
CBHC	All services provided by people who spend a substantial part of their working time outside a health facility, discharging their services at the individual, family or community level as well as primary healthcare services provided in small local health facilities (World Health Organization, 2016)
Post-conflict	A transitional period, characterized by destabilization, where past war or conflict exists on one end and a future period of peace on the other, often most associated with a period of rebuilding and reconstruction

Only study designs reporting a specific outcome related to healthcare were included. Studies without empirical data, conference abstracts, posters or protocols were excluded from the review. We followed close definitions of key terms in determining eligibility of studies (Box 1). For example, a study was excluded from the review if the study author(s) did not explicitly include details of conflict, crisis or war as a feature of the context of the study; such studies were excluded even when the reviewers were aware that conflict had existed in the area. Likewise, CHWs were defined as a cadre of healthcare providers who are (1) from the community they serve and (2) without formal training (Lehmann and Sanders, 2007). For example, midwives have become an increasingly professionalized cadre in recent years, and they often receive formal training (Castro Lopes *et al.*, 2016). Therefore, they were considered to be formal healthcare workers for this review, and articles that discussed delivery of care by midwives were excluded. Similarly, care strategies that focused on the delivery of services within a clinical setting, regardless of size or remoteness, were not considered ‘community-based’ and were excluded from this review.

Duplicate studies were eliminated using Microsoft Excel (Microsoft Corporation, Redmond, WA, USA) (Microsoft Corp, 2019). Following PRISMA guidelines, two reviewers independently assessed studies for eligibility first by title and abstract, removing those that did not meet the criteria (Liberati *et al.*, 2009). Full texts of the remaining articles were then retrieved and screened again using the inclusion criteria. Reviewers checked all within-publication references to identify additional sources. As a desk-based review, no ethical approval was sought.

### Data extraction and analysis

Data were drawn from included full-text articles and compiled using a predefined 13-item extraction sheet. Reviewers then used the matrix to summarize descriptively the results in tables by country of origin, study design, condition or disease addressed, type of intervention and effect size or impact of the intervention. Descriptive analysis of key characteristics of included publications was conducted, and consensus on themes related to the key functions of CHWs was reached by dialogue between the two reviewers.

## Results

**Figure 1.**
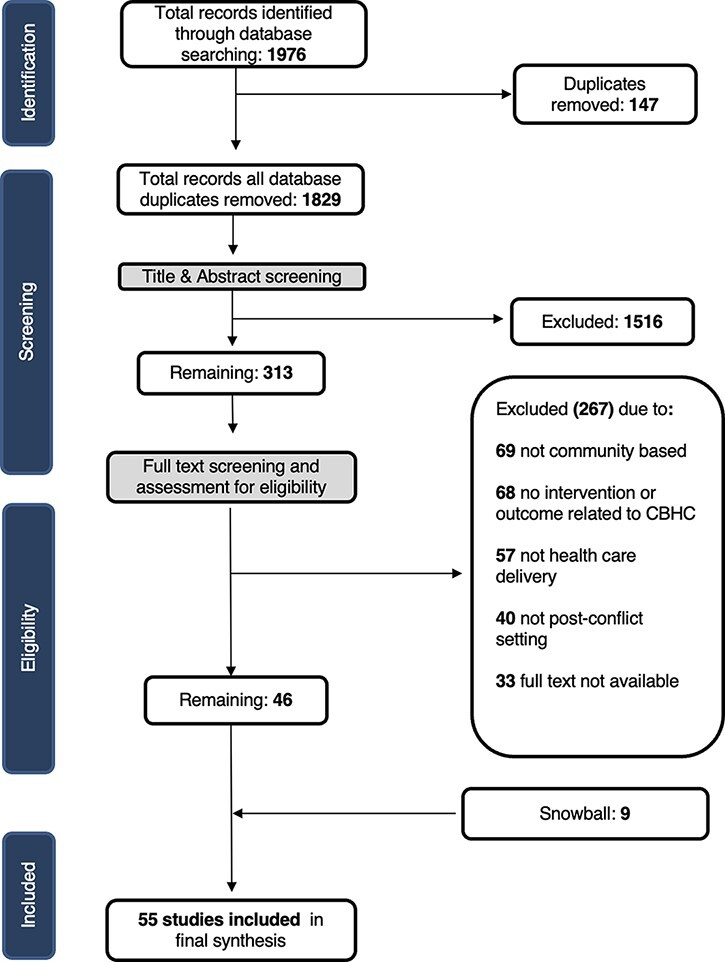
PRISMA diagram of included studies

A total of 1976 studies were identified in our search (Figure 1); 147 duplicates were removed. Based on title and abstract review, 1516 studies were excluded. Full-text screening for eligibility was conducted on 313 articles: 68 studies did not assess community-based intervention or CHWs and were removed, 57 were not related to the delivery of healthcare, 49 did not report a specific outcome connected to CHW or CBHC and 40 were not conducted in post-conflict settings. Of the 313 articles, 33 studies either had no full text available or were available only as an abstract or protocol, and 15 of the 313 studies were systematic reviews with no empirical outcome. Although these studies were not included in our review, their reference lists were searched. Snowball methods were used to search reference lists for all studies included after full-text review, adding nine papers. The process resulted in 55 full-text articles included in our final review.

### Study characteristics

The final synthesis included representation from 19 countries across four regions. Liberia was the most frequently reported country (*n = *11), followed by Afghanistan (*n = *10) and then Uganda (*n = *8). Over half of all the studies (*n = *35) reported were derived from the sub-Saharan African region, with the remainder from the South Asian region (*n = *17), the East Asian Pacific Region (*n = *5) and the Middle East North African region (*n = *4). Of 55 studies, 11 were conducted in rural settings and 2 in urban settings (Weiss *et al.*, 2015). Three articles focused specifically on displaced persons, refugee camps or humanitarian settings (Bolton *et al.*, 2007; Shanks *et al.*, 2013; Naal *et al.*, 2021).

The majority of the articles used an observational study design (*n = *30). These included cross-sectional studies (*n = *19) (Edward *et al.*, 2007; Hadi *et al.*, 2007; Ahmadzai *et al.*, 2008; Teela *et al.*, 2009; Huber *et al.*, 2010; Mullany *et al.*, 2010; Viswanathan *et al.*, 2012; Shanks *et al.*, 2013; Mayhew *et al.*, 2014; Nanyonjo, 2014; Luckow *et al.*, 2017; Ratnayake *et al.*, 2017; Ruckstuhl *et al.*, 2017; Soe *et al.*, 2017; Edmond *et al.*, 2018; Kohrt *et al.*, 2018; Rogers *et al.*, 2018; Kasang *et al.*, 2019; Dal Santo *et al.*, 2020), case studies (*n = *6) (Marsh *et al.*, 2012; Giugliani *et al.*, 2014; Newbrander *et al.*, 2014; Ventevogel, 2016; Brault *et al.*, 2018; Healey *et al.*, 2021), longitudinal studies (*n = *3) (Smith *et al.*, 2014a,b; Oo, 2018) and cohort studies (*n = *2) (Hawkes *et al.*, 2009; Wickett *et al.*, 2018). Of the final 55 studies, 12 studies were controlled trials (Bass *et al.*, 2012; Dawson *et al.*, 2018; Mutamba *et al.*, 2018; White *et al.*, 2018; Schneider *et al.*, 2020), including 7 randomized control trials (Bolton *et al.*, 2007; Weiss *et al.*, 2015; Bass *et al.*, 2016; Rahman *et al.*, 2016; 2019a,b; Nakimuli-Mpungu *et al.*, 2020). Six of the studies were qualitative studies utilizing survey methodologies, focus groups or interviews (Bass *et al.*, 2013; Abramowitz *et al.*, 2015; Fiekert, 2002; Musinguzi *et al.*, 2017; Zou *et al.*, 2020; Naal *et al.*, 2021). Six studies used mixed methods(Palmer *et al.*, 2014; Khan *et al.*, 2017; Ojanduru *et al.*, 2018; Mai *et al.*, 2019; Van Boetzelaer *et al.*, 2019; Ferdinand *et al.*, 2020) and one used a simulation model (Stijntjes, 2015). Additional details are shown in Table 2 (See Annexe).

**Table 2.** Key characteristics of included studies

Author (year)	Country origin of data	Methods/study design	Disease area	Intervention
Abramowitz *et al.* (2015)	Liberia	Qualitative study	Infectious diseases	Community-based triage training
Ahmadzai *et al*. (2008)	Afghanistan	Cross-sectional	Infectious diseases	DOTS
Bass *et al*. (2016)	Iraq	RCT	Mental health	Psychoeducation
Bass *et al*. (2012)	Indonesia	Controlled trial	Mental health	Problem-solving counselling
Bass *et al*. (2013)	Democratic Republic of Congo	Qualitative study	Mental health	Psychotherapy
Boetzelaer *et al*. (2019)	South Sudan	Mixed methods	Malnutrition	Treatment protocol and screening for malnutrition
Bolton *et al*. (2007)	Uganda	RCT	Mental health	Psychotherapy
Brault *et al*. (2018)	Liberia	Case study	Maternal and child health	Outreach campaigns, CHWs and trained traditional midwives
Dal Santo *et al*. (2020)	Afghanistan	Cross-sectional	Reproductive health	Computer tablet-based health video library with counselling
Dawson *et al*. (2018)	Indonesia	Controlled trial	Mental health	Cognitive behaviour therapy
Edmond *et al*. (2018)	Afghanistan	Cross-sectional	Maternal and child health	CHW household visits
Edward *et al*. (2007)	Mozambique	Observational	Childhood illness	CHW household visits
Ferdinand *et al*. (2020)	Central African Republic	Mixed methods	Infectious diseases	Case management
Fiekert (2002)	Afghanistan	Qualitative study	Infectious diseases	DOTS
Giugliani *et al*. (2014)	Angola	Case study	Maternal and child health	Placing CHWs in the community
Hadi *et al*. (2007)	Afghanistan	Cross-sectional	Reproductive health	CHW household visits
Hawkes *et al*. (2009)	Democratic Republic of Congo	Cohort study	Infectious diseases	Rapid diagnostic testing
Healey *et al*. (2021)	Liberia	Case study	Maternal and child health	CHW household visits
Huber *et al*. (2010)	Afghanistan	Cross-sectional	Reproductive health	CHW household visits
Kasang *et al*. (2019)	Liberia	Cross-sectional	Infectious diseases	Case identification
Khan *et al*. (2017)	Pakistan	Mixed methods	Mental health	At-home psycho-educational sessions
Kohrt *et al*. (2018)	Uganda, Liberia and Nepal	Cross-sectional	Mental health	Training and supervision programme for CHWs
Luckow *et al*. (2017)	Liberia	Cross-sectional	Maternal and child health	CHW household visits
Mai *et al*. (2019)	Democratic Republic of Congo	Mixed methods	Reproductive health	Community-based family planning
Marsh *et al*. (2012)	Sierra Leone	Case study	Childhood illness	iCCM
Mayhew *et al*. (2014)	Afghanistan	Cross-sectional	Malnutrition	Community-based growth monitoring sessions
Mullany *et al*. (2010)	Burma (Myanmar)	Cross-sectional	Reproductive health	CHW household visits
Musinguzi *et al*. (2017)	Uganda	Qualitative study	Primary health care	Village health teams
Mutamba *et al*. (2018)	Uganda	Controlled trial	Mental health	Training for village caregivers
Naal *et al*. (2021)	Lebanon	Qualitative study	Reproductive health	Female CHWs
Nakimuli-Mpungu *et al*. (2020)	Uganda	Randomized controlled trial	Mental health	Group support psychotherapy
Nanyonjo (2014)	Uganda	Cross-sectional	Childhood illness	iCCM
Newbrander *et al*. (2014)	Afghanistan	Case study	Maternal and child health	Basic package of health services
Ojanduru *et al*. (2018)	Uganda	Mixed methods	Reproductive health	Community-based group learning and counselling
Oo (2018)	Afghanistan	Longitudinal study	Maternal and child health	iCCM
Palmer *et al*. (2014)	South Sudan	Mixed methods	Infectious diseases	Screening and referrals
Rahman *et al*. (2016)	Pakistan	Randomized controlled trial	Mental health	Weekly individual sessions on problem solving, behavioural activation and stress management
Rahman *et al*. (2019b)	Pakistan	Randomized controlled trial	Mental health	Group sessions on behavioural strategies
Rahman *et al*. (2019a)	Pakistan	Randomized controlled trial	Mental health	Tablet-based training application and cascaded supervision
Ratnayake *et al*. (2017)	Sierra Leone	Cross-sectional	Childhood illness	iCCM
Rogers *et al*. (2018)	Liberia	Cross-sectional	Infectious diseases	Community-based adherence support
Ruckstuhl *et al*. (2017)	Central African Republic	Cross-sectional	Infectious diseases	At-home case management
Schneider *et al*. (2020)	Uganda	Controlled trial	Mental health	Narrative exposure therapy delivered by lay counsellors
Shanks *et al*. (2013)	CAR, Colombia, DRC, India, Iraq, Pakistan, Papua New Guinea and Russia	Cross-sectional	Mental health	Routine mental health programme with individual counselling
Smith *et al*. (2014a)	Liberia	Longitudinal study	Reproductive health	Providing education and misoprostol to pregnant women
Smith *et al*. (2014b)	South Sudan	Longitudinal study	Reproductive health	Distribution of misoprostol during home visits
Soe *et al*. (2017)	Myanmar	Cross-sectional	Infectious diseases	Community mobilization and awareness raising
Stijntjes (2015)	Liberia	Simulation model	Infectious diseases	Disease surveillance
Teela *et al*. (2009)	Burma (Myanmar)	Cross-sectional	Reproductive health	Lay maternal health workers placed in the community
Ventevogel (2016)	Burundi	Case study	Mental health	Psychosocial volunteers
Viswanathan *et al*. (2012)	Afghanistan	Cross-sectional	Reproductive health	Contraceptives
Weiss *et al*. (2015) [19]	Iraq	Randomized controlled trial	Mental health	Cognitive processing therapy
White *et al*. (2018) [54]	Liberia	Controlled trial	Childhood illness	iCCM
Wickett *et al*. (2018) [50]	Liberia	Cohort study	Infectious diseases	Food support, reimbursement of transport and social assistance
Zou *et al*. (2020) [64]	Sierra Leone	Qualitative study	Chronic diseases	Hypertensive and diabetic case management

DOTS = directly observed treatment strategy.

Interventions described in the articles addressed several disease areas. The most frequently reported disease area was mental health and psychosocial well-being (*n = *16) (Bolton *et al.*, 2007; Bass *et al.*, 2012; 2013; 2016; Shanks *et al.*, 2013; Weiss *et al.*, 2015; Ventevogel, 2016; Khan *et al.*, 2017; Dawson *et al.*, 2018; Kohrt *et al.*, 2018; Mutamba *et al.*, 2018; Rahman *et al.*, 2019a,b; Nakimuli-Mpungu *et al.*, 2020; Schneider *et al.*, 2020; Bowsher *et al.*, 2021) followed by maternal and reproductive health (*n = *12) (Hadi *et al.*, 2007; Teela *et al.*, 2009; Huber *et al.*, 2010; Mullany *et al.*, 2010; Viswanathan *et al.*, 2012; Smith *et al.*, 2014a,b; Edmond *et al.*, 2018; Ojanduru *et al.*, 2018; Mai *et al.*, 2019; Dal Santo *et al.*, 2020; Naal *et al.*, 2021), infectious diseases (*n = *12) (Ahmadzai *et al.*, 2008; Hawkes *et al.*, 2009; Palmer *et al.*, 2014; Abramowitz *et al.*, 2015; Fiekert, 2002; Stijntjes, 2015; Ruckstuhl *et al.*, 2017; Soe *et al.*, 2017; Rogers *et al.*, 2018; Wickett *et al.*, 2018; Kasang *et al.*, 2019; Ferdinand *et al.*, 2020) and childhood illnesses (*n = *11) (Edward *et al.*, 2007; Marsh *et al.*, 2012; Giugliani *et al.*, 2014; Nanyonjo, 2014; Newbrander *et al.*, 2014; Luckow *et al.*, 2017; Ratnayake *et al.*, 2017; Brault *et al.*, 2018; Oo, 2018; White *et al.*, 2018; Healey *et al.*, 2021).

Group psychosocial strategies were the most commonly reported intervention for mental health and psychosocial well-being (*n = *6) and evaluated amongst diverse populations. Five studies evaluated continuity of mental healthcare through various means, including the provision of routine mental health services across nine humanitarian settings (Shanks *et al.*, 2013); home-based psychosocial educational sessions (Khan *et al.*, 2017); integrating psychiatric care into general healthcare services (Ventevogel, 2016) and the use of non-specialist mental health provision in primary care settings (Kohrt *et al.*, 2018; Rahman *et al.*, 2019b) (Rahman *et al.*, 2016). Our review included additional interventions such as treatment of post-traumatic stress disorders (Bass *et al.*, 2016; Schneider *et al.*, 2020), behavioural psychotherapy counselling approaches (Weiss *et al.*, 2015) and the use of technology-assisted trainings to scale up trained CHWs (Rahman *et al.*, 2019a).

Six studies investigated maternal and reproductive health strategies covering antenatal and post-natal care interventions (Edmond *et al.*, 2018), which aimed to increase reproductive services awareness (Hadi *et al.*, 2007), emergency obstetrics care (Mullany *et al.*, 2010), contraceptive use (Huber *et al.*, 2010; Mai *et al.*, 2019) and family planning (Ojanduru *et al.*, 2018). Other interventions identified in our review included advanced distribution of misoprostol (Smith *et al.*, 2014a; 2014b); the use of health video libraries for community counselling (Dal Santo *et al.*, 2020); a combined programme of modern contraceptive use, antenatal care and skilled birthing attendance (Viswanathan *et al.*, 2012) and a mobile obstetric maternal health programme (Teela *et al.*, 2009; Mullany *et al.*, 2010).

Many of the childhood illness studies evaluated integrated community case management (iCCM) strategies (Marsh *et al.*, 2012; Nanyonjo, 2014; Ratnayake *et al.*, 2017; White *et al.*, 2018). Nearly all studies found large substantive improvements—as indicated by statistical analysis—in treatment by qualified providers and decreased under-five mortality; although, two of the studies found no significant changes for diarrhoea treatment (Nanyonjo, 2014; Ratnayake *et al.*, 2017). Three studies investigated the impact of outreach campaigns conducted by CHWs (Edward *et al.*, 2007; Giugliani *et al.*, 2014; Brault *et al.*, 2018). These studies reported expanded access to care and improved referrals, which resulted in reductions in under-five mortality and infant mortality. Other interventions reported efforts to strengthen basic health services (Newbrander *et al.*, 2014; Healey *et al.*, 2021) and case management for combined maternal and child health programming (Luckow *et al.*, 2017; Oo, 2018; Healey *et al.*, 2021).

Leveraging communities for epidemic control efforts were explored in two papers, and evidence indicated that CHWs were more timely than professional healthcare data entry clerks and played a key role in community triage for Ebola patients (Abramowitz *et al.*, 2015). As such, they may be well suited to report outbreaks in near real time (Stijntjes, 2015).

Two studies evaluated the capacity of task sharing to CHW cadres with lower literacy levels to deliver care specifically related to nutrition care. Simplified treatment protocols strategies were found to improve weight-for-age scores (Mayhew *et al.*, 2014), average diastolic blood pressure after hypertension and diabetes diagnosis (Zou *et al.*, 2020) and performance checklists use (Van Boetzelaer *et al.*, 2019).

### Key functions of CHWs

Our review identified evidence regarding four key functions of CHWs in healthcare delivery in post-conflict settings including (1) access to care and treatment coverage, (2) case management and adherence support, (3) disease detection and monitoring and (4) scaling up of services. Further details of the types of strategies reported for each function, which reported measurable impacts, are provided in Table 3 (see Annexe).

**Table 3.** Reported impact outcomes by CHW key health care delivery function

Author (year)	Intervention	Impact
Access to care and treatment coverage		
Ahmadzai *et al.* (2008)	Directly observed treatment (DOTS) for TB	+135% treatment coverage
Abramowitz *et al.* (2015)	Community-based epidemic control strategies for Ebola	+ access to care
Bass *et al.* (2012)	Group psychotherapy	No effect on burden of depression and anxiety symptoms + in positive coping strategy use
Bass *et al.* (2013)	Group psychotherapy	-Decreased PTSD, depression and anxiety symptoms
Bass *et al.* (2016)	Group psychotherapy	-Lowered scores for anxiety and depressive symptoms
Edmond *et al*. (2018)	Community health home visits	−66% in infant mortality −62% in under-five mortality
Hadi *et al.* (2007)	Female CHW presence in community	+53.9% coverage of women receiving antennal care +10.3% women receiving tetanus toxoid injections
Huber *et al.* (2010)	CHW presence in community	+10% increased use of contraceptives
Musinguzi *et al.* (2017)	CHWs as link to formal healthcare services	No reported outcomes
Nanyonjo (2014)	iCCM	+34.7% children receiving antibiotics for pneumonia +41% receiving oral rehydration solutions for diarrhoea
Ojanduru *et al.* (2018)	Community-based group learning and counselling	+ in knowledge of reproductive control methods
Rogers *et al.* (2018)	Community-based treatment and social assistance	HIV/AIDS: +3.8% antiretroviral therapy treatment coverage TB: +35.5% treatment coverage
Schneider *et al.* (2020)	Trauma-focused cognitive behavioural therapy	-decreased PTSD symptoms on self-reported score (−9.26) and caregiver-reported measures (3.53) -symptom reduction (−26.41)
Soe *et al.* (2017)	Community-based TB care	Contributed to detection of 36% of total new TB cases in respective townships
Smith *et al.* (2014a, b)	Advance distribution of misoprostol	+20% more likely for women to ingest misoprostol at correct time + increased coverage of misoprostol use
Case management and adherence support		
Ahmadzai *et al.* (2008)	Directly observed treatment (DOTS) for TB	+86% treatment success
Rogers *et al.* (2018)	Community-based treatment and social assistance	HIV/AIDS: +22.2% patient retention and −6% LTFU TB: −7.5% LTFU
Marsh *et al.* (2012)	iCCM	−69% of severe pneumonia cases −21% to −52% under-five mortality
Ruckstuhl *et al.* (2017)	CHW case management	98.9% of positive malaria cases appropriately treated
White *et al.* (2018)	iCCM	+ childhood disease treatment by qualified provider + correct diarrhoeal treatment
Disease detection and monitoring		
Hawkes *et al.* (2009)	Rapid diagnostic testing	+ identification of malaria in febrile children
Kasang *et al.* (2019)	Training CHW to identify suspected cases of leprosy	+25% new cases reported −6.2% disability rate of new cases
Mayhew *et al.* (2014)	Community growth monitoring	+ weight-for-age scores by 0.3
Palmer *et al.* (2014)	Train CHWs to recognize potential syndromic cases of HAT during routine outpatient practice	+ appropriate referrals
Stijntjes *et al.* (2015)	CHW smart phone-based data entry for disease surveillance	+ timeliness of detecting disease outbreak
Zou *et al.* (2020)	Training CHWs to improve diagnosing NCDs	+ average diastolic blood pressure of hypertensive/diabetic patients by 8 mmHg
Scaling up services		
Abramowitz *et al.* (2015)	Community-based epidemic control strategies	+ access to care
Dal Santo *et al.* (2020)	Tablet-based health video library	+ patients seeking reproductive maternal and child health counselling
Huber *et al.* (2010)	CHW presence in community	+10% increased use of contraceptives
Kohrt *et al.* (2018)	Training non-specialists to integrate mental health care into primary care	+ in demonstrated knowledge
Rahman *et al.* (2019a)	Tablet-based training application and cascaded supervision to train CHWs	No change reported
Van Boetzelaer *et al.* (2019)	Simplified treatment protocol for CHWs	+ 2% improvement in malnutrition checklist completion

+ indicates increase; − indicates decrease; HAT = human African trypanosomiasis; LTFU = loss to follow-up; NCDs = noncommunicable diseases; TB = tuberculosis; PTSD = post-traumatic stress disorder.

### Access to care and treatment coverage

Fifteen studies reported outcomes related to access to care and treatment coverage and revealed that CHW interventions are highly successful at reducing treatment gap for populations in hard-to-reach conflict-affected areas. Many interventions were both feasible and successful in raising awareness among communities, which in turn may improve access to care, increase treatment coverage and link and connect communities to formal healthcare services (Hadi *et al.*, 2007; Musinguzi *et al.*, 2017; Soe *et al.*, 2017; Ojanduru *et al.*, 2018). Studies found improved coverage of care, measured by an increase in the number of patients receiving care services. This improvement in access was particularly notable in studies focused on services for reproductive and maternal health and psychosocial support.

Of particular significance, several studies suggest that CHW interventions were able to provide mental healthcare for individuals residing in FCAPCS, where residents are especially prone to post-traumatic stress. For example, one study evaluated the impact of a trauma-informed support, skills and psychoeducation intervention provided by CHWs in northern Iraq on depressive symptoms, post-traumatic stress and anxiety (Bass *et al.*, 2016). Study results revealed that the intervention has a statistically significant and moderate-sized effect on depression symptoms and a small effect on post-traumatic stress and anxiety.

### Case management and adherence support

Nine studies in our review identified the benefits of CHW case management and supportive care in improving health behaviours of the population. CHWs contributed to routine clinical care and treatment adherence support approaches such as directly observed treatment (Ahmadzai *et al.*, 2008; Fiekert, 2002; Rogers *et al.*, 2018; Wickett *et al.*, 2018) and case management (Ruckstuhl *et al.*, 2017; Ferdinand *et al.*, 2020). In all instances, the percentage of patients receiving treatment increased, while patient attrition or loss to follow-up dropped dramatically.

For example, one study found that CHWs trained in case management resulted in a significant increase in the percentage of children receiving care from formal care providers (Luckow *et al.*, 2017). More specifically, the study found that care for diarrhoea increased by 60 percentage points, care for fever increased by 31 percentage points and care for acute respiratory infection increased by 51 percentage points. Adherence support delivered by CHWs was found to decrease loss to follow-up in HIV patients by 6 percentage points and increase patient retention of ART treatment by 22 percentage points (Rogers *et al.*, 2018). In Liberia, one study found loss to follow-up rates decreased by 76% as a result of accompaniment assistance to patients with TB (Wickett *et al.*, 2018); this change is highly impactful as TB is highly curable with uninterrupted antituberculosis therapies.

### Disease detection and monitoring

Eight studies reported on disease detection and monitoring efforts for infectious diseases. Our review found evidence that CHWs lead to more widespread appropriate referrals for Gambiense-type human African trypanosomiasis (Palmer *et al.*, 2014) and malaria (Ferdinand *et al.*, 2020). Similar results were noted for improvements in new case detection of leprosy (Kasang *et al.*, 2019). Using CHWs to actively search and identify cases and provide referrals was found to increase in new case detection (Soe *et al.*, 2017; Kasang *et al.*, 2019), lead to more widespread appropriate referrals (Palmer *et al.*, 2014) and raise community awareness of disease (Soe *et al.*, 2017).

The advantage of engaging CHWs was also evident in disease surveillance efforts. For example, one study showed that CHWs are likely to detect outbreaks in a timelier fashion than untrained public health data enterers (Stijntjes, 2015). Concomitantly, CHWs trained to screen and identify disease were then able to link communities to more formal care.

### Scaling up services

CHWs were found to contribute to the scaling up of healthcare services in two major ways. The first is by means of their location and integration into the community in hard-to-reach areas, making it easier for them to provide faster care. This pattern is highlighted by Stijntjes (2015); CHWs have the capacity to conduct near-real-time disease surveillance quicker than professional data enterers (Stijntjes, 2015). In addition, Abramowitz *et al*. (2015) reported similar results, noting that locally engaged community members were able to address absences of infrastructure and material support in order to contain the Ebola epidemic in their communities (Abramowitz *et al.*, 2015).

The second mechanism through which CHWs contributed to scaling up of health services was through task shifting and task sharing. Of studies included in this review, four evaluated CHWs’ abilities to share healthcare tasks with formally trained workers (Mayhew *et al.*, 2014; Stijntjes, 2015; Van Boetzelaer *et al.*, 2019; Zou *et al.*, 2020). In all cases, CHWs were found to excel in providing healthcare tasks, or surveillance, that are normally provided by formally trained workers. CHWs were determined to be useful, particularly in instances of uncomplicated care, such as certain cases of malnutrition or other common instances of care. For example, in Afghanistan, one study found that community-based growth monitoring and promotion—delivered by lower literacy female CHWs—improved children’s weight-for-age scores by 0.3 standard deviations from the mean (Mayhew *et al.*, 2014).

## Discussion

This systematic review described and summarized the body of literature pertaining to CHW healthcare delivery in fragile and conflict-affected settings. To our knowledge, this is the first study to identify systematically the evidence of CHWs’ impact on enhancing healthcare and rebuilding the health system after a period of conflict. Studies indicated that community-based efforts may address key barriers to delivering care in the context of disrupted health systems by facilitating access to care, strengthening disease detection and improving adherence to care. Our results align with the broader literature on the success of CHWs and point to a particular value of these interventions in the context of post-conflict settings.

We identified studies that point to the value of leveraging CHWs to deliver healthcare and serve the unique health needs of populations residing in fragile and conflict-affected settings. Studies indicate that CHWs could (1) increase access to essential healthcare services, (2) improve case management and treatment adherence, (3) enhance disease detection and monitoring and (4) facilitate the scaling up of services.

Evidence suggests that CHW interventions are successful at addressing the treatment gap for populations in hard-to-reach conflict-affected areas. More specifically, CHWs were able to provide mental healthcare for individuals residing in FCAPCS, where residents are especially prone to post-traumatic stress. Health outcomes are improved with adherence to treatment regimens, which require consistent access to medicines and can be complicated by the ongoing insecurity in FCAPCS. Engaging a network of CHWs, who are already embedded in the community, to collect health information and report disease outbreaks may lead to earlier detection and better monitoring where health information infrastructure may be severely weakened. CHWs may be an effective resource in detecting new cases of infectious diseases.

Despite fractured health systems in FCAPCS, scalable CHW interventions were documented to improve the availability of services and accessibility to care. Task shifting, or task sharing, from more specialized healthcare workers to a broader base of CHWs can directly ameliorate supply-side issues. As such, task shifting and sharing protocols may allow CHWs to specialize in specific tasks where they have comparative advantage, thus freeing up more time for formally trained health workers to complete more complicated tasks.

iCCM interventions, which train and deploy CHWs to hard-to-reach areas, are frequently implemented in many low- and middle-income countries (Guenther *et al.*, 2014). This systematic review adds to the literature by identifying and including four studies (out of 55) on the effect of iCCM interventions in fragile states and conflict-affected areas. These studies found that the iCCM intervention increased (1) treatment by qualified provider, (2) coverage of appropriate treatment of fevers and (3) the proportion of children with pneumonia who received antibiotics and oral rehydration salts among children with diarrhoea. Another observed benefit was improved quality of services, measured both by patient perception and adherence to guidelines, as a result of delivering more appropriate care (Nanyonjo, 2014; Ratnayake *et al.*, 2017; Oo, 2018). With 39.8% of those living in FCAPCS under the age of 15, childhood diseases appear to particularly benefit from case management by CHWs (World Health Organization, 2017).

The findings on the effectiveness of iCCM in fragile states and conflict-affected settings are promising and should be considered in conjunction with an alternative form of case management, Proactive community case management (ProCCM). ProCCM interventions add to iCCM by including active case detection, doorstep care and monthly dedicated supervision by CHWs as well as the removal of user fees and inclusion of primary care infrastructures (Johnson *et al.*, 2018). Future research should systematically evaluate, compare and contrast the effect of iCCM and ProCCM on ameliorating a range of different public health concerns.

However, literature from non-conflict settings indicate serious challenges sustaining these observed gains as a result of access issues. In Malawi, perception of CHWs as convenient and high-quality deteriorated over time with caregivers preferred providers other than CHWs (Amouzou *et al.*, 2016). At the end of the study, less than half of sick children were brought to CHWs (Zalisk *et al.*, 2019). A better understanding of the sustainability of iCCM in post-conflict settings is necessary.

Decentralizing care during times of instability and unrest was a fundamental part of all included studies. By delivering care at a community level, and thus dispersing the delivery of care outside a hospital setting, it was possible to manage disease in hard-to-reach areas and among populations that have suffered from displacement due to conflict. Highly scalable technologies could improve the feasibility of increasing the number of trained CHWs across the challenges of a fragile and conflict-affected setting—such as distance, a limited number of trainers and limited budgets for training expenses. In one such example of applying scalable technologies, Rahman *et al*. (2019b) found that cascading training structures using tablet-based applications were as effective at training CHWs as face-to-face training (Rahman *et al.*, 2019a).

One study identified in our review cautioned that lack of clear delineation of roles risks an ambiguity between the role of CHWs and formal healthcare. Roles should be carefully thought out and clearly delineated for community members (Musinguzi *et al.*, 2017).

### Key enablers for CHW system success

Box 2. Key enablers contributing to the success of CHW interventions

**Table:** 

Training and support	Material resources included in intervention
Training to increase knowledge for human African trypanosomiasis [Marsh *et al.*, 2012]	Technology for case management [Dal Santo *et al.*, 2020]
Trauma-informed support skills [Bass *et al.*, 2016]	Free of charge services and resources [Mai *et al.*, 2019; Nanyonjo 2014]

There is some indication that challenges faced by CHWs in fragile and conflict-affected settings are somewhat similar to the challenges that many CHWs face worldwide (Miller *et al.*, 2020). Recent systematic reviews of CHW programmes indicate that the success of CHW systems hinges on a number of important factors (Perry and Zulliger, 2012; Kok *et al.*, 2015a; Scott *et al.*, 2018). A sample of key enablers identified by this study is highlighted in Box 2. These enablers broadly can be grouped into two categories: training and support provided to CHWs and material resources provided to CHWs. Examples of training and support that contributed to the success of an intervention included training CHWs to increase their knowledge in disease conditions [e.g. human African trypanosomiasis (Marsh *et al.*, 2012), leprosy (Kasang *et al.*, 2019) and noncommunicable diseases (Zou *et al.*, 2020)] and improving the care provided [e.g. trauma-informed care (Bass *et al.*, 2016)]. Material resources included integrating technology in the care process [e.g. computer tablet-based health video library (Dal Santo *et al.*, 2020)] and offering free care [e.g. contraception (Nanyonjo, 2014; Mai *et al.*, 2019)]. Other factors include integration into the health system and community participation and acceptance (Scott *et al.*, 2018).

While evidence suggests that these enablers contributed to CHWs’ ability to provide better care than without these enablers, it is critical to note that achieving these factors can be particularly challenging in conflict and post-conflict settings. For example, existing health systems and community trust are often broken in fragile contexts, and training logistics can be challenging in the face of delayed supplies (Amouzou *et al.*, 2016). A detailed discussion on the barriers that impede last mile service falls beyond the scope of this review; however, the literature suggests that both supply and demand side barriers may impede CHW programmes such as training, management, supervision, funding, political support, alignment with existing healthcare providers and acceptability to the community (Pallas *et al.*, 2013).

Evidence also suggests that individuals with more formal education preceding their CHW role may be more effective (Kok *et al.*, 2015b), while education is often disrupted in fragile contexts, resulting in lower literacy levels for potential CHWs (Raven *et al.*, 2020). Although appropriate selection criteria of CHWs are undetermined, consideration should be given to attributes such as age, gender, literacy level, language skills and residency within the community, which could determine CHWs’ effectiveness (Lehmann and Sanders, 2007; Jaskiewicz and Deussom, 2013). None of the studies included in our review measured the impact of renumeration of CHWs; however, evidence indicates that renumeration policies may influence motivation and subsequent success of programme goals (Witter *et al.*, 2012).

Finally, management and supervision of CHWs also play a critical role in determining intervention success, yet several challenges such as travelling long distances across damaged infrastructure, periods of restricted travel and security threats limit this capacity in FCAPCS (Miller *et al.*, 2020). Therefore, it is important for health planners to recognize what intervention design factors are and are not feasible within the parameters of post-conflict settings.

Future research could benefit from comparing and contrasting strategies (e.g. iCCM versus ProCCM) and evaluating their effectiveness. Furthermore, included studies mainly assessed outcomes of quality of care or improved coverage; thus, they only implied rather than directly reported on health benefits. Studies with more straightforward outputs that precisely measure the potential improvements to disease morbidity and mortality should be considered. This endeavour could be accomplished through the use of rigorous randomized control trials to assess, for example, reductions in disability or death as a result of CHW interventions. Our review contained limited data on the cost and cost-effectiveness of CHW interventions. However, post-conflict settings may be able to apply some of the lessons from research conducted in fragile settings such as Mozambique (Bowser *et al.*, 2015) or crisis-affected settings (Witter *et al.*, 2017).

### Limitations

By including a range of study designs, we were unable to use statistical methods to compare these different types of results. The results of our study are also vulnerable to publication bias in favouring positive results, although two studies reported no positive changes after intervention (Bass *et al.*, 2012; Ratnayake *et al.*, 2017). We defined the scope of our search to the best of our ability, but with no consistent global usage of terms used in our search, such as ‘fragile state’, ‘post-conflict’ and ‘community-based healthcare’, it is possible that additional studies exist that did not make our final list. For example, terms such as ‘lay health worker’ were not used, and therefore, some studies may have been missed.

## Conclusion

Evidence suggests that CHW interventions may be not only effective but also efficient in circumventing the barriers associated with access to care in fragile states and conflict-affected areas and improve health outcomes. The main themes observed in the literature included improvements in treatment coverage, increased disease detection rates and better health outcomes. Case management strategies (e.g. iCCM) were documented to be effective in improving a wide range of health outcomes. Furthermore, task-sharing strategies have been emphasized as a common mechanism for incorporating CHWs into health systems.

The results of this review indicate that policies should leverage the strengths of CHW programmes to achieve better health outcomes. However, these policies should ensure that varying CHW programmes and interventions are considered while recognizing that each conflict-affected setting is unique. One-size-fits-all policies should be considered with caution.

## Supplementary Material

czac072_Supp

## Abbreviations

CBHC = community-based healthcare

CHW = community health workers

FCAPCS = fragile, conflict-affected, and post-conflict states

HAT = human African trypanosomias

iCCM = integrated community case management

LTFU = loss to follow-up

NCD = noncommunicable diseaseTB = tuberculosis

## Reflexivity Statement

The authors include three females and one male and span multiple levels of seniority. Our paper covers a variety of these regions, including those to which named authors are affiliated. While three authors specialize in health, nutrition and population in the Middle East North African and South Asian regions, the fourth specializes in emergency care systems in the sub-Saharan African region. All authors have experience conducting policy and quantitative research in these regions.
