# Supplementary material for: The role of community health worker-based care in post-conflict settings: a systematic review
Source: Health Policy Plan. 2022 Sep 19;38(2):261–74. doi: 10.1093/heapol/czac072 (PMC9923383; doi:10.1093/heapol/czac072)
Supplement: czac072_Supp [file czac072_supp.zip › Supplementary 1.docx]

**Supplementary 1. Search Strategy**

| **DATABASE (n)** | **SEARCH STRINGS** |
| --- | --- |
| OVID MEDLINE (30) | 1 ((community health) adj2 (worker? or professional? or personnel or manpower or staff or workforce or volunteer?)).tw.  2 ((community based or community-based or community level) adj2 (care or approach or approaches or delivery or healthcare or health delivery or healthcare delivery)).tw.  3 (community driven or community care service? or community networks or community participation or home care service?).tw.  4 or/1-3  5 ((post-conflict* or combat or fragile) adj2 (area? or zone? or setting? or region or regions or military or armed or ethnic or country or countries or state or states or field? or recovery)).tw.  6 (post-conflict? or postconflict? or post-war? or postwar? or (post adj (war or wars or conflict?))).tw.  7 (health adj (system? or service?) adj (reconstruction or reconstructing or rehabilitation or rehabilitating or rebuilding)).tw.  8 or/5-7  9 4 and 8  10 limit 9 to yr="2000 -Current" |
| Embase (14) | 1 (‘community health’) next/2 (‘worker?’ or ‘professional?’ or ‘personnel’ or ‘manpower’ or ‘staff’ or ‘workforce’ or ‘volunteer?’)  2 (‘community based’ or ‘community-based’ or ‘community level’) next/2 (‘care’ or ‘approach’ or ‘approaches’ or ‘delivery’ or ‘healthcare’ or ‘health delivery’ or ‘healthcare delivery’)  3 ‘community driven’ or ‘community care service?’ or ‘community network?’ or ‘community participation’ or ‘home care service?’  4 #1 OR #2 OR #3  5 (‘post-conflict*’ or ‘combat’) next/2 (‘area?’ or ‘zone?’ or ‘setting?’ or ‘region?’ or ‘military’ or ‘armed’ or ‘ethnic’ or ‘country’ or ‘countries’ or ‘state?’ or ‘field?’ or ‘recovery’)  6 ‘post-conflict?’ or ‘postconflict?’ or ‘post-war?’ or ‘postwar?’ or (‘post’ next/2 (‘war?’ or ‘conflict?’ or ‘zone?’))  7 ‘fragile’ next/2 (‘country’ or ‘countries’ or ‘region?’ or ‘state?’ or ‘setting?’)  8 ‘health’ next/2 (‘system?’ or ‘service?’) next/2 (‘reconstruction’ or ‘reconstructing’ or ‘rehabilitation’ or ‘rehabilitating’ or ‘rebuilding’)  9 #5 OR #6 OR #7 OR #8  10 #4 AND #9  11 #10 AND [2000-2021]/py |
| CINHAL (63) | S11 S4 AND S10  S10 S5 OR S6 OR S7 OR S8 OR S9  S9 TX ((health N1 (service* or system*)) N1 (reconstruction or reconstructing or rehabilitation or rehabilitating or rebuilding))  S8 TX (fragile N1 ( area or areas or zone or zones or setting* or region or regions or country or countries or state or states)  S7 TX (post N1 conflict*)  S6 TX (post-conflict* or postconflict* or post-war* or postwar*)  S5 TX ((post-conflict* or post-combat) N2 (area or areas or zone or zones or setting* or region or regions or military or armed or ethnic or country or countries or state or states or field or fields or recovery))  S4 S1 OR S2 OR S3  S3 TX (community driven or community care service* or community network* or community participation or home care service*)  S2 TX ((community based or community-based or community level) N2 (care or approach or approaches or delivery or healthcare or health delivery or healthcare delivery))  S1 TX ((community health) N2 (worker* or professional* or personnel or manpower or staff or workforce or volunteer*)) |
| SCOPUS (653) | TITLE-ABS-KEY ({community health worker} OR {community health workers} OR {community health volunteer} OR {community health volunteers} OR {community health workforce} OR {community health personnel} OR {community health manpower} OR {community health staff} OR {community health professional} OR {community based care} OR {community based approach} OR {community based approaches} OR {community based delivery} OR {community based healthcare} OR {community based health delivery} OR {community based healthcare delivery} OR {community-based care} OR {community-based approach} OR {community-based approaches} OR {community-based delivery} OR {community-based healthcare} OR {community-based health delivery} OR {community-based healthcare delivery} OR {community level care} OR {community level healthcare approach} OR {community healthcare approaches} OR {community level health approach} OR {community health approaches} OR {community level healthcare} OR {community level health delivery} OR {community level healthcare delivery} OR {community driven health} OR {community care service} OR {community care services} OR {homecare services})  AND  TITLE-ABS-KEY({post-conflict} OR {postconflict} OR {post-war} OR {postwar} OR {post-conflict area} OR {post-conflict zone} OR {post-conflict state} OR {post-conflict states} OR {post-conflict regions} OR {fragile state} OR {fragile states} OR {conflict recovery} OR {health system reconstruction} OR {health system rehabilitation} OR {health system reconstructing} OR {health system rehabilitating} OR {health system rebuilding} OR {health service reconstruction} OR {health service rehabilitation} OR {health service reconstructing} OR {health service rehabilitating} OR {health service rebuilding})  AND PUBYEAR AFT 2000 |
| Web of Science (44) | TS= (“community health worker” OR “community health workers” OR “community health volunteer” OR “community health volunteers” OR “community health workforce” OR “community health personnel” OR “community health manpower” OR “community health staff” OR “community health professional” OR “community based care” OR “community based approach” OR “community based approaches” OR “community based delivery” OR “community based healthcare” OR “community based health delivery” OR “community based healthcare delivery” OR “community-based care” OR “community-based approach” OR “community-based approaches” OR “community-based delivery” OR “community-based healthcare}” OR “community-based health delivery” OR “community-based healthcare delivery” OR “community level care” OR “community level approach” OR “community level approaches” OR “community level delivery” OR “community level healthcare” OR “community level health delivery” OR “community level healthcare delivery” OR “community driven” OR “community care service” OR “community care services” OR “homecare services” OR “community participation” OR “community pharmacy services”)  AND  TS= (“post-conflict” OR “post conflict” OR “post-conflict setting” OR “postconflict setting” OR “post-conflict country” OR “postconflict country” OR “post-conflict countries” OR “post conflict countries” OR “post-conflict zone” OR “post conflict zone OR post-conflict region” OR “post conflict region” OR “post-war” OR “postwar” OR “post-war setting” OR “postwar setting OR “post-war state” OR “postwar state” OR “post-war country” OR “postwar country” OR “post-war countries” OR “postwar countries” OR “post-war zone” OR “postwar zone” OR “post-war region” OR “postwar region” OR health system reconstruction” OR “health service reconstruction” OR “fragile setting” OR “fragile state” OR “fragile states” OR “fragile nation” OR “fragile nations” OR “fragile country” OR “fragile countries” OR “fragile region” OR “fragile regions” OR “conflict recovery”) |
| Cochrane (21) | MeSH:  Community Health Services, Community Health Workers  OR  Keywords:  ((“community health”) next (“worker?” or “professional?” or “personnel” or “manpower” or “staff” or “workforce” or “volunteer?”)) or (“community based” or “community-based” or “community level”) next (“care” or “approach” or “approaches” or “delivery” or “healthcare” or “health delivery” or “healthcare delivery”) or “community care service?” or “home care service?”  AND  MeSH:  Armed conflicts  OR  Keywords:  "post-conflict?" or "postconflict?" or "post-war?" or "postwar?" or ("post" next (“war?” or “conflict?” or “zone?”))  (“post-conflict*” or “combat”) next (“area?” or “zone?” or “setting?” or “region?” or “military” or “armed” or “ethnic” or “country” or “countries” or “state?” or “field?” or “recovery”)  “fragile” next (“country” or “countries” or “region?” or “state?” or “setting?”)  “health” next (“system?” or “service?”) next (“reconstruction” or “reconstructing” or “rehabilitation” or “rehabilitating” or “rebuilding”) |
| Google Scholar (1172) | "Community based health service" OR "Community-based health service" OR "community based health care" OR "community-based health care" OR "community health worker" AND "post conflict" OR "post war" OR "post-war" OR "post-conflict" |
